# Changes in the Structure and Digestibility of Wrinkled Pea Starch with Malic Acid Treatment

**DOI:** 10.3390/polym10121359

**Published:** 2018-12-07

**Authors:** Miaomiao Shi, Qunyu Gao, Yanqi Liu

**Affiliations:** 1 School of Food and Biological Engineering, Zhengzhou University of Light Industry, Zhengzhou 450002, China; liuyanqi@zzuli.edu.cn; 2 Collaborative Innovation Center of Food Production and Safety, Zhengzhou 450002, China; 3 Henan Key Laboratory of Cold Chain Food Quality and Safety Control, Zhengzhou 450002, China; 4 Carbohydrate Laboratory, School of Food Science and Engineering, South China University of Technology, Guangzhou 510640, China; qygao@scut.edu.cn

**Keywords:** wrinkled pea starch, malic acid, ^13^C CP/MAS NMR, esterification reaction

## Abstract

Resistant starch has gradually become a popular food component due to its beneficial physiological effects and heat resistance during processing. In this study, the structure, reaction mechanism, and digestibility of wrinkled pea starch with malic acid and heat–moisture treatment (HMT) are investigated. The degree of substitution (DS) of malate starch, HMT-malate starch, and malate-HMT starch was 0.164, 0.280, and 0.146, respectively. Malate starch remained in its complete particle form and pronounced birefringence was displayed. However, the malate-HMT starch sample was almost completely broken into pieces and lost the polarized cross. All modified starch samples had a decreased swelling power and a new peak at 1731–1741 cm^−1^ shown by FTIR. From the ^13^C CP/MAS NMR (Cross Polarizatio/Magic Angle Spinning Nuclear Magnetic Resonance) spectra, all the modified starches had extra peaks at 38.5 ppm and 172.8 ppm. After esterification treatment, the resistant starch (RS) and slowly digestible starch (SDS) content of starch samples increased dramatically. The higher content of RS and lower enzymatic hydrolysis rate of the malate starch could be used to produce low-calorie foods and have potential health benefits.

## 1. Introduction

Starch is the main glycemic carbohydrate material in cereal- and tuber-based food products [1]. According to the rates of digestion, starch can be classified into different groups: rapidly digestible starch (RDS), slowly digestible starch (SDS), and resistant starch (RS). RS cannot be absorbed in the small intestine of healthy individuals, and hence, might be fermented in the colon [2]. The processing method and the state of the starch determine the digestibility of the starch [3]. The purpose of the modification of starch is to improve its properties and potential application value [4]. Due to its beneficial physiological effects, RS has been promoted in recent years as a new functional nutritional ingredient in the development of original food products [5].

Recently, many researchers have supported numerous methods to prepare RS from different sources. They mainly include physical [6,7], chemical [8], and enzymatic [9] modification or the combinations of any two methods [10,11]. However, the most effective way to increase resistant starch content is through chemical modification. Several studies have shown that the esterification reaction can significantly increase the resistant starch content [12,13]. In the field of food, the citrated starch obtained from the esterification reaction or crosslinking reaction between citric acid and starch could be used as a safe reacting agent [14,15,16]. Malic acid is also a safe and nontoxic food additive and its structure and function are similar to citric acid. Malic acid belongs to the polycarboxylic acids and has a lower melting point and decomposition point than citric acid. Therefore, in theory, malic acid should be more easily esterified or crosslinked with starch. To improve the reaction efficiency between starch molecules and malic acid, heat–moisture treatment (HMT) is occasionally used.

Pea is an important source of starch and is grown worldwide as one of the oldest domesticated food crops. China is the second largest producer of peas in the world (1.6 million tonnes (MT)) followed by Russia (1.4 MT), USA India (600 kilotonnes (KT)), and USA (7.4 KT). China’s pea production is second only to Canada (3.4 MT) [17]. Depending on the different seed phenotypes, peas are divided into two kinds: smooth and wrinkled peas. The surface of smooth peas is smooth, similar to soybeans and mung beans. However, the surface of wrinkled peas is usually pleated, wrinkled, and not smooth. The two types of peas are genetically different, so the starches have different structural and functional characteristics. Since smooth pea starch has a high resistant starch content and slower digestion rate, it can play a better functional role in the food field [18,19,20]. Therefore, there have been many reports on the modification of smooth pea starch. The effects of alkali on the structure and function of smooth pea starch were investigated on the basis of limited gelatinization of the granules [21]. Smooth pea starch and dextrin polymers were modified through the unequal reactivity of isocyanate groups in isophorone diisocyanate (IPDI) monomers [22]. However, researchers have produced relatively few studies on the structure and properties of wrinkled peas, particularly Chinese wild varieties. The structure and digestibility of wrinkled pea starches are the subjects of the present study. In this study, we explored the reaction mechanisms of wrinkled pea starch and malic acid to produce RS, and simultaneously investigated the effect of HMT on the properties of esterified starch.

## 2. Materials and Methods

### 2.1. Materials

Wrinkled pea (a wild variety cultivated in China) was obtained from Dingxi Gansu Province. Pancreatin from porcine pancreas (P7545, 8 × USP) and amyloglucosidase from *Aspergillus niger* (A7095, 300 U/mL) were purchased from Sigma-Aldrich Chemical Co. (St. Louis, MO, USA). Glucose oxidase–peroxidase (GOPOD) assay kit was obtained from Megazyme International Ireland Ltd. (Wicklow, Ireland). Chemicals and solvents were of an analytical grade.

### 2.2. Starch Isolation and Synthesis of Malate Derivatives of Wrinkled Pea Starch

Starch was extracted from wrinkled pea seeds by the method of Beta et al. [23]. The obtained wrinkled pea starch contained 30.8% amylose, 0.37% protein, 0.13% fat, and 0.14% ash content.

Malate starch was prepared according to the method of Klaushofer et al. [24] with slight modifications. Wrinkled pea starch (30 g dry weight) was added into a malic acid solution (20% *w*/*w*) to produce a 25% suspension by weight, and the pH was adjusted to 3 with 10 mol/L NaOH. The mixture was dried at 45 °C to the moisture content of 5–10% (*w*/*w*) after the starch suspension was thoroughly infiltrated for 24 h at room temperature. The mixture was then finely ground and put into a hydrothermal synthesis reactor (model F4-100, Zhengzhou Brocade Instrument Equipment Co. Ltd., Zhengzhou, China) for 6 h at 140 °C. The unreacted malic acid was removed by washing several times. The obtained starch was dried at 40 °C and powdered. The sample was denoted as malate starch. 

Malate starch (20 g dry weight) was weighed into hydrothermal synthesis reactors. Starch moisture content was brought to 25% by adding the appropriate amount of distilled water. The hydrothermal reactors were sealed and then placed in a forced-air oven at 120 °C for 12 h. Then, starches were washed once with deionized water and dried in an oven at 40 °C, ground, and passed through a 100-mesh screen. The sample was denoted as malate-HMT starch. 

Similarly, the native starch was brought to 25% moisture content at 120 °C for 12 h to produce HMT starch, and then the sample was reacted with malic acid according to the above method and was denoted as HMT-malate starch.

### 2.3. Scanning Electron Microscopy (SEM)

Starch samples were covered with 20 nm of gold under vacuum by double-sided adhesive tape fastened to a round aluminum stub. Starch granules were observed and photographed at 1500× magnification with a scanning electron microscope (model TM3000, Hitachi, Tokyo, Japan).

### 2.4. Polarized Light Microscopy

Birefringence of starch granules was viewed with an optical microscope (BHS-2, Olympus, Tokyo, Japan). Each sample was disseminated in solution (glycerine/water; 1:1 *v*/*v*), and the images were taken at 500× magnification.

### 2.5. Determination of Degree of Substitution (DS)

The DS of starch samples was established using a titration technique [25]. The starch samples (5.0 g dry weight) were precisely weighed and disseminated with 50 mL distilled water in a conical flask. Then, phenolphthalein solution (1% *w*/*w*) was included as an indicator. The suspension was modified to be a reddish solution, and then 25 mL of NaOH (0.45 mol/L) was placed into the flask by stirring for 60 min. After that, the starch solution was titrated with a 0.2 mol/L standard HCl solution. The native starch was utilized as the control. The DS was determined with the following Equation:

(1) $$A\left(\%\right)=\frac{\left(V_{0}-V_{1}\right)\times c\times M\times 100}{W\times 1000}$$

(2) $$DS=\frac{162\times A}{100M-\left(M-1\right)A}=\frac{162\times A}{11700-116A}$$

where V_0_ is the consumed volume of hydrochloric acid standard solution of the blank, V_1_ is the consumed volume of hydrochloric acid standard solution of the starch sample, A is the mass fraction of malate substituents (%), c is the standard molarity of the HCl solution, W is the weight of starch, M is the molecular weight of 2-hydroxy-succinyl (117), and 162 is the relative molecular mass of glucosyl.

### 2.6. Swelling Power and Starch Solubility

The starch slurry (2% *w*/*v*) was heated in a shaking water bath at 65, 80, and 95 °C, respectively, for 30 min, and then quickly chilled to room temperature. After centrifugation at 4000 r/min for 15 min, the supernatant was dried at 105 °C for 2 h and the remainder was weighed. The weight of the dried supernatant divided by the weight of the dry starch was the solubility (%). The weight of the remainder divided by the weight of the dry starch was the swelling power (g/g).

### 2.7. Fourier-Transform Infrared Spectroscopy (FTIR)

FTIR was determined based on the technique of Li et al. [26]. Each of the infrared spectra was acquired on a Vector 33 spectrometer attachment (Bruker, Karlsruhe, Germany) with a resolution of 4 cm by 64 scans. KBr was mixed with starch in the ratio of 1:15. Spectra were baseline-corrected by creating a straight line between 4000 and 400 cm^−1^. Each starch sample was drenched in a relative humidity chamber overnight at ambient temperature prior to FTIR determination.

### 2.8. Nuclear Magnetic Resonance Spectroscopy (NMR)

Solid-state ^13^C CP/MAS NMR analyses of starch samples were performed using a AVANCE III HD 400 MHz NMR spectrometer (Bruker, Germany) using the method of Ye et al. [27] with some modifications. The dried samples were packed in the 4-mm ZrO_2_ rotor closed with a Kel-F cap and were spun at a rate of 8 kHz. The adoption time was 17 min and the delay time was 2 s. The spectral width was 295.8 ppm. Samples were analyzed at ambient temperature. 

### 2.9. In Vitro Digestion with Pancreatin and Amyloglucosidase

Digestion properties were examined utilizing the technique of Englyst et al. [2] with alterations. The enzyme solution was prepared by mixing 0.75 mL of amyloglucosidase (300 U/mL) and 2.25 g pancreatin (8 × USP) in sodium acetate buffer (7.5 mL, 0.1 M, pH 5.0). Starch (200 mg) was dispersed in 10 mL of sodium acetate buffer (0.1 M, pH 5.0), and then 0.75 mL of enzyme solution (the mixture of amyloglucosidase and pancreatin) was added. Enzyme digestion took place in a 37 °C water bath at 150 rpm, and 0.5 mL aliquots of hydrolyzed solution were gathered at different time intervals. The hydrolyzed solution was subjected to enzyme deactivation using ethanol (20 mL, 95%). After centrifugation (1500× *g*, 10 min), the glucose amount was established utilizing a GOPOD assay kit. The RDS, SDS, and RS amounts were acquired by utilizing the following formulae:

RDS (%) = 90 × (G20 − FG)/TS(3)

SDS (%) = 90 × (G120 − G20)/TS(4)

RS (%) = 100 − RDS% − SDS%(5)

### 2.10. Statistical Analysis

The variations among the mean values of multiple groups were evaluated by SPSS 17.0 and Origin 8.0 software for one-way analysis of variance (ANOVA) with Duncan’s multiple-range tests. ANOVA data with a *p* value of <0.05 were established as being statistically significant. Mean values were obtained from triplicate experiments. 

## 3. Results and Discussion

### 3.1. Granule Structure and DS of Starch Samples

**Figure 1:**
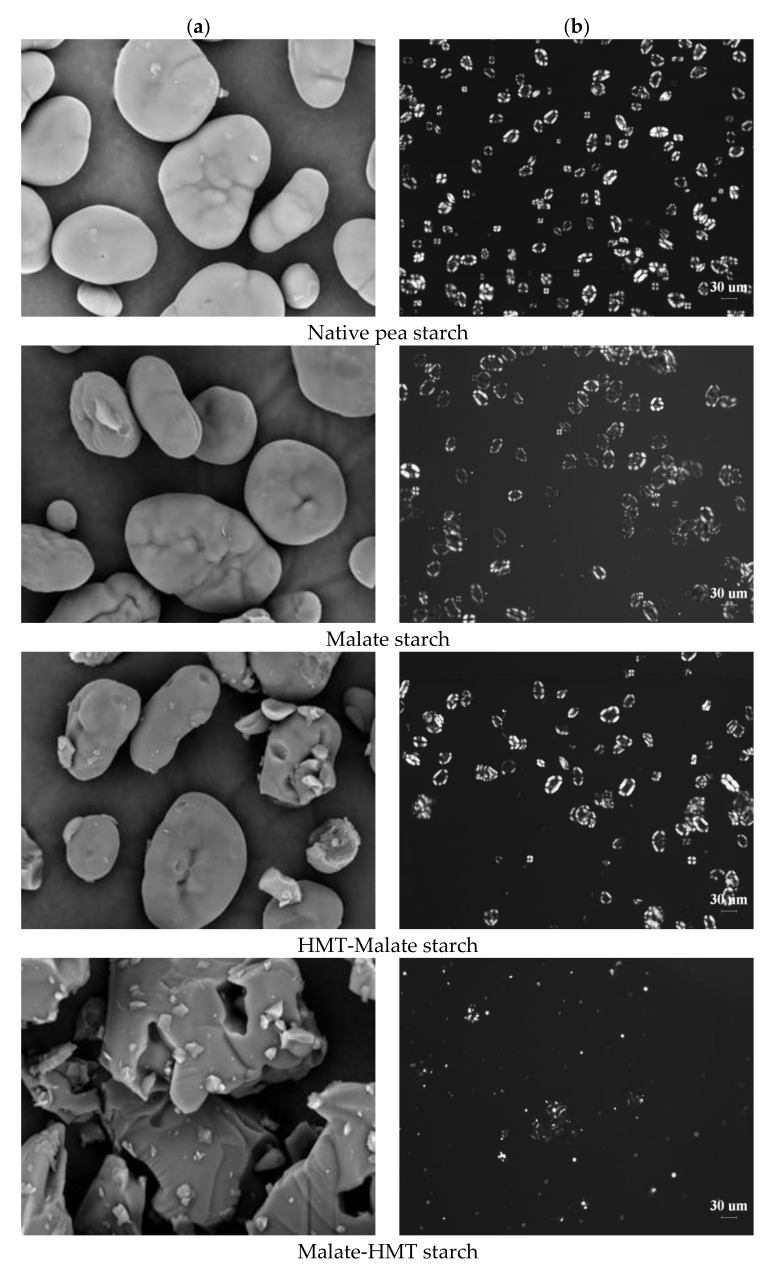
SEM images (1500×) (**a**) and birefringence images (**b**) of malate starch samples. HMT: heat–moisture treatment.

Figure 1a gives SEM micrographs and the DS of native and modified starches. Native wrinkled pea starches have different sizes of granules. The large granules appear oval or round, and the small granules appear spherical. The surfaces of the starch granules were smooth and had no fissures or ruptures. Most of the granules have a smooth surface. Some of the larger starch granules had slight indentations and grooves, which were proven to be typical of legume starch granules. This may be due to protein residues on the starch granule surface, which made the pure starch from the pea seed difficult to obtain [28,29]. For malate starch, most starch granules remained intact and some of the granules had deep indentations and grooves. A portion of the HMT-malate starch sample still had the intact granule form, and the granules of the other portion were broken. However, the malate-HMT starch sample was almost completely broken into pieces. It can be seen that the hydrothermal treatment had some effect on the esterification of the starch. Especially after the formation of esterified starch, hydrothermal treatment had a greater impact on the granule shape. From the values of DS, it was found that a greater degree of substitution did not correspond to the greater crushing of particles. The DS of malate starch, HMT-malate starch, and malate-HMT starch was 0.164, 0.280, and 0.146, respectively. This may be due to HMT not substantially altering the granule shape and size of the native starch granules [7,30]. The DS of HMT-malate starch was higher than that of malate starch, indicating that the structure of starch after hydrothermal treatment was more conducive to the esterification reaction. However, the granule structure of the malate-HMT starch sample was severely damaged and the DS was slightly decreased compared to the malate starch. This may be due to the change in the internal structure of the starch after the esterification reaction. Under high-temperature conditions, hydrothermal treatment might easily destroy the sample. These results indicated that the esterification reaction changed the starch granule properties [14].

Birefringence is a symbol of the average radial orientation of helical structures [26]. At the center of the native starch, as shown in Figure 1b, pronounced birefringence was displayed. A clearly recognizable “Maltese cross” pattern was shown in native starch granules under polarized light. The birefringence strength might be impacted by granule shape and the position of the granule in relation to the light beam [31]. For the malate starch, the polarized cross still existed and the intensity of birefringence was slightly lower. For the HMT-malate starch, the intensity of the birefringence demonstrated almost no changes and the polarized cross also still existed. However, the polarized cross of the malate-HMT starch sample no longer existed. These results were consistent with the results of SEM.

### 3.2. Swelling Power and Starch Solubility

**Figure 2:**
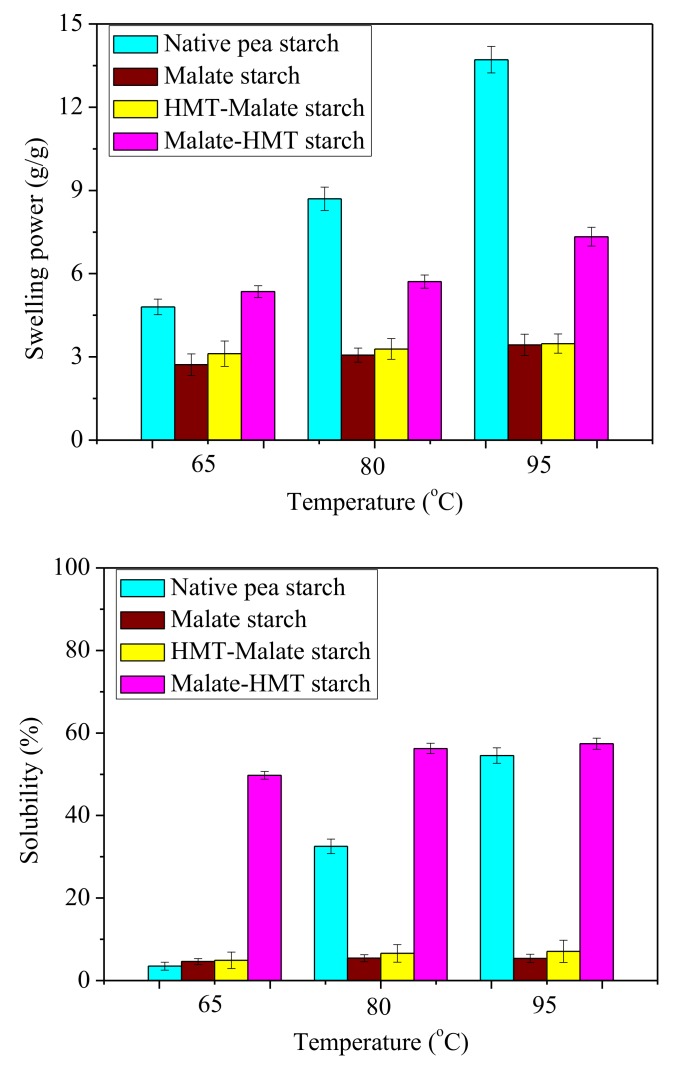
Swelling power and solubility of malate starch samples.

Swelling power and solubility properties of the native and modified samples are shown in Figure 2. The swelling power of starch granules demonstrated the amalgamation scope of starch chains inside the amorphous and crystalline domains. During the gelatinization of the starch granules, the amorphous region firstly swelled and then slowly spread to the crystallization region [32]. As shown by Figure 2, the swelling power of all starches increased as the temperature increased from 65 °C to 95 °C. Compared with the native pea starch, all malic acid-modified starch samples had a decreased swelling power. This could be due to the fact that the accessible OH groups in the starch polymer were esterified and were no longer accessible to the water molecules. This made it more difficult for the water molecules to penetrate into the starch. Malate starch and HMT-malate starch had a similar swelling power. However, the malate-HMT starch had the highest swelling power. This was consistent with the structural damage of starch granules. As the temperature increased, the solubility of the starch increased. This suggested that the crystal structure of the starch successively disappeared in the process of heating, and that the hydrogen bonding of water had a tendency to combine hydroxyl groups of amylose [32]. Malate starch and HMT-malate starch had a similar decrease in swelling power; however, malate-HMT starch had a higher swelling power compared with native pea starch. This may be related to its low degree of substitution. A similar increase in solubility after heat–moisture treatment was observed for these starch samples that received malic acid and heat–moisture treatment. Depending on the different reaction conditions, starch and malic acid may undergo a substitution reaction, and a crosslinking reaction also may occur [14,33]. Therefore, the solubility and swelling power of starch samples were reduced compared with the native pea starch.

### 3.3. FTIR of Starch Samples 

**Figure 3:**
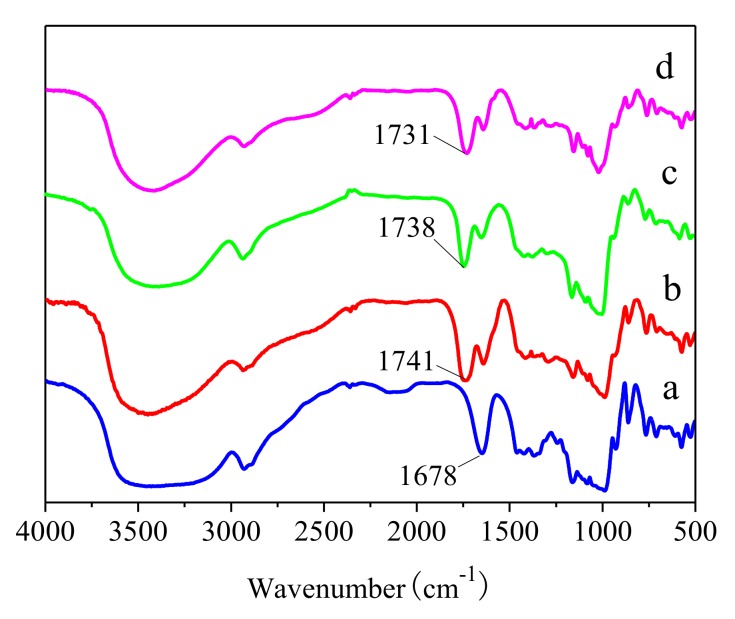
FTIR spectra of malate starch samples: (**a**) Native pea starch, (**b**) malate starch, (**c**) HMT-malate starch, and (**d**) malate-HMT starch.

FTIR spectroscopy can reflect changes in the chemical structure of starch molecules. The FTIR spectra of native pea starch and modified starch samples were shown in Figure 3. A new peak at 1731–1741 cm^−1^ appeared in all modified samples, but it was not observed in their control. The band at 1731–1741 cm^−1^ was associated with the stretching of the C=O bond from the ester group. This suggested the successful esterification of starch with malic acid. Compared with the native starch, the malic acid-treated pea starch showed a decrease in the intensity of peaks at 1630–1650 cm^−1^ (aromatic ring stretch), which was related to the transformation of the C–O bond.

### 3.4. ^13^C CP/MAS NMR of Starch Samples

**Figure 4:**
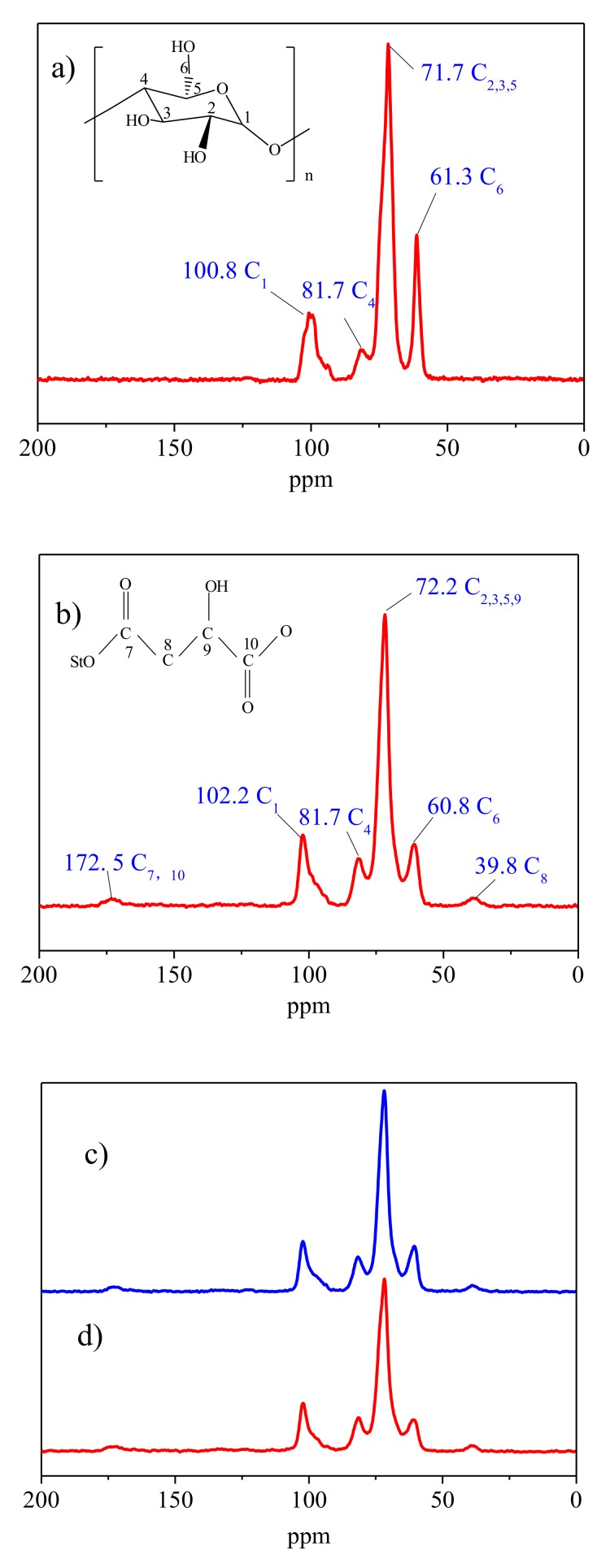
^13^C CP/MAS NMR spectra of malate starch samples: (**a**) native pea starch, (**b**) malate starch, (**c**) HMT-malate starch, and (**d**) malate-HMT starch.

The ^13^C CP/MAS NMR spectra of the native wrinkled pea starch and modified starch samples are shown in Figure 4. In the literature, it has been noted that the peaks of C_1_ usually appear at 94–105 ppm; the resonances of C_2_, C_3_, and C_5_ overlap at 68–78 ppm; and peaks at 80–84 ppm and 58–65 ppm are linked to C_4_ and C_6_ sites, respectively [34]. The C_2_, C_3_, and C_5_ sites had the similar chemical environment in soiled ^13^C NMR spectra, so they were covered by a broad signal peak [35,36]. From Figure 4, all the modified starches had the extra peaks in the ^13^C NMR spectrum compared with the native pea starch. The peaks at 35–50 ppm were correlated to the hybridized carbon atom units (–(CH_2_–CH)_n_) in the polymers [37]. Carbon resonances of the ester group (170–175 ppm) were documented in the spectrum of modified starch [38]. This suggested the successful esterification of starch with malic acid. This was consistent with the results of FTIR. The resonance at 38.5 ppm was attributed to C_8_ sites, and resonance at 172.8 ppm was associated with C_7_ and C_10_ sites. Since the carbon atom on the ester group and the carboxyl group were bonded to the oxygen atom in the form of a double bond, the two resonance peaks were not distinguishable from the ^13^C NMR spectrum. In addition, the C_9_ site had a similar chemical environment to the C_2_, C_3_, and C_5_ sites, so they were covered by a broad signal peak.

**Figure 5:**
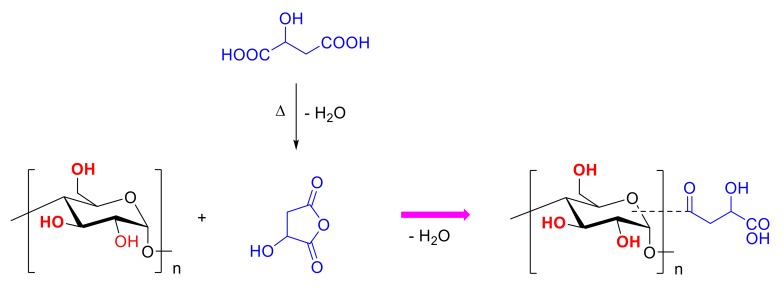
Reaction equation of starch and malic acid.

Compared with the native starch, the C_1_ chemical shifts of the malate starch moved downfield by 1.4 ppm, while the chemical shifts of the other carbons also had a little change (C_2,3,5_, 0.5 ppm; C_4_, 0.0 ppm; C_6_, 0.5 ppm). This could not enable the definition of which hydroxyl group of the carbon atom had the esterification reaction. It is well known that dehydration of hydroxyl groups at C_1_ and C_4_ of the glucose rings generally forms glycosidic bonds. Therefore, the esterification reaction was difficult to induce in the C_1_ site. However, the C_1_ chemical shift of the malate starch was bigger than for the other carbon atoms. This may be due to the steric hindrance caused by esterification groups. Kapelko-Zeberska et al. [39] investigated the molecular structure changes of starch citrate by NMR and HPSEC (High Performance Size Exclusion Chromatography). These researchers found that intermolecular crosslinking of the dextrin produced by acid hydrolysis caused the starch molecular chains to link together and produce a larger molecular weight amylopectin. Studies have shown that that the reaction should occur both in the amorphous phase and partial crystalline phase [14]. It was concluded that malic acid preferentially reacted with amylopectin. HMT-malate starch and malate-HMT starch had a similar ^13^C NMR spectra compared to malate starch. From the results of FTIR spectroscopy and the ^13^C CP/MAS NMR spectra, Figure 5 predicted the reaction progress of starch and malic acid.

### 3.5. The Sample Contents of the Three Nutritional Components

The contents of RDS, SDS, and RS of the starch samples before and after cooking are shown in Table 1. For the native pea starch, the contents of the three nutritional components before and after cooking showed a comparatively large change. RDS content changed from 7.91% to 88.99% after cooking. RS content decreased from 84.69% to 4.07%. SDS content of the starch samples before and after cooking showed almost no change. Cooking under high temperature transformed the RS of the native pea starch granules to RDS. After esterification treatment, the RS content of starch samples increased dramatically in comparison with the control. Vu et al. [40] also reported that HMT could be used to increase the RS content in sorghum flour without gelatinizing its starch. Especially, malate starch and HMT-malate starch had a higher RS content, which reached 70%. This may be related to their high degree of substitution. Due to the steric hindrance of the substituents on the starch chains, the attack of the enzyme was prevented, resulting in the inability of the starch-digesting enzyme to completely hydrolyze the starch [8,15]. At elevated levels of substitution, the enzyme vulnerability was reduced. Simultaneously, the contents of three nutritional components of malate starch and HMT-malate starch after cooking showed only a little change. This showed that the two samples were resistant to high temperatures. However, malate-HMT starch had a lower RS content (~40%) and a higher SDS content (~20%) than the above two esterified starch samples. The decreased RS content of malate-HMT starch might be due to the molecular rearrangement caused by the heat–moisture treatment, which disrupted the ester bonds between malic acid and starch. This was consistent with its low degree of substitution. The higher content of RS and lower enzymatic hydrolysis rate of the malate starch could be used to produce low-calorie foods and have potential health benefits [15].

## 4. Conclusions

This study showed that malic acid and heat–moisture treatment can be used to decrease the enzyme susceptibility and digestion properties of wrinkled pea starch. Malate starches can still maintain particle integrity. The application of heat–moisture treatment prior to the esterification reaction could help to improve the degree of substitution of the starch products. Both FTIR spectroscopy and the ^13^C CP/MAS NMR spectra analysis showed the formation of new peaks that indicated that the esterification of starch and malic acid was carried out. The ^13^C CP/MAS NMR spectra showed that the hydroxyl groups participated in the reaction and predicted the reaction progress of starch and malic acid. Each of the outcomes suggested that HMT applied before the esterification reaction raised the availability of the starch granules and made it simpler for malic acid to infiltrate the starch granules. This caused the RS and SDS content of the starch samples to increase dramatically in comparison with the native starch, and also reduced enzyme sensitivity. Based on the heat-resistant stability of the resistant starch and the uniform and delicate appearance, it is expected that the resistant starch can be well applied in the field of food processing.

## Figures and Tables

**Table 1:** Percentages (% *w*/*w*, dry weight) of rapidly digestible starch (RDS), slowly digestible starch (SDS), and resistant starch (RS) ^†^.

Starch Sample	Uncooked			Cooked		
	RDS (%)	SDS (%)	RS (%)	RDS (%)	SDS (%)	RS (%)
Native pea starch	7.91 ± 0.53 a	7.40 ± 0.28 a	84.69 ± 0.81 d	88.99 ± 2.01 c	6.94 ± 0.66 a	4.07 ± 2.67 a
Malate starch	13.90 ± 0.20 b	14.87 ± 0.55 b	71.23 ± 0.75 c	14.52 ± 0.61 a	12.45 ± 0.21 b	73.03 ± 0.82 d
HMT-malate starch	13.37 ± 0.43 b	16.33 ± 0.14 c	70.30 ± 0.57 b	13.85 ± 1.33 a	15.43 ± 0.71 c	70.72 ± 2.04 c
Malate-HMT starch	36.44 ± 0.52 c	22.78 ± 0.33 d	40.78 ± 0.19 a	38.83 ± 0.46 b	20.34 ± 0.40 d	40.83 ± 0.06 b

^†^ Values with a different letter in the same column are significantly different (*p* < 0.05).
